# Brain allopregnanolone induces marked scratching behaviour in diet-induced atopic dermatitis mouse model

**DOI:** 10.1038/s41598-019-38858-3

**Published:** 2019-02-20

**Authors:** Masanori Fujii, Sayaka Ohgami, Erika Asano, Takanori Nakayama, Takahiro Toda, Takeshi Nabe, Susumu Ohya

**Affiliations:** 1 Department of Pharmacology, Division of Pathological Sciences, Kyoto Pharmaceutical University, 5 Nakauchi-cho, Misasagi, Yamashina, Kyoto 607-8414 Japan; 2 Laboratory of Immunopharmacology, Faculty of Pharmaceutical Sciences, Setsunan University, 45-1 Nagaotoge-cho, Hirakata, Osaka 573-0101 Japan; 3 Department of Pharmacology, Graduate School of Medical Sciences, Nagoya City University, 1 Kawasumi, Mizuho-cho, Mizuho, Nagoya 467-8601 Japan

## Abstract

Allopregnanolone (ALLO) is a neurosteroid produced in the brain, but so far, no study has explored its link with itching. Herein, we used a diet-induced atopic dermatitis mouse model to examine whether exogenously administered and endogenously produced ALLO contribute to inducing scratching. Systemic administration of ALLO elicited robust scratching in the atopic dermatitis model, while it did not affect spontaneous and pruritogen-induced scratching in normal mice. ALLO caused scratching when administered intracisternally, but not when administered intrathecally or intradermally, suggesting the involvement of supraspinal mechanisms. Pharmacological analyses suggested that both γ-aminobutyric acid type A receptor activation and serotonin type 3 receptor inhibition were involved in ALLO-induced scratching. We next examined whether endogenously produced ALLO is involved in ethanol-induced scratching in atopic dermatitis mice, because ethanol administration increases ALLO in rodent brain. Acute ethanol administration increased brain ALLO levels, which coincided with increased scratching. Pre-treatment with finasteride, a synthetic ALLO inhibitor, suppressed ethanol-induced scratching and ALLO production in the brain. Collectively, our results demonstrated for the first time that ALLO administration caused marked scratching in atopic dermatitis mice, and ethanol-induced scratching may be mediated through endogenously produced brain ALLO.

## Introduction

Itch (or pruritus) is an unpleasant sensation inducing the desire to scratch. Atopic dermatitis is a common chronic skin disease, and pruritus is a cardinal symptom of this disease, which markedly reduces the quality of life of the patient^1^. Although several pathogenic mechanisms in the periphery and spinal cord have been postulated to be involved in atopic dermatitis itch^2–5^, supraspinal (i.e. brain) mechanisms may also play an important role. It is known that clinically, emotional stress, sleep, and alcohol intake often trigger or enhance pruritus in atopic dermatitis^6^, and these factors seem to primarily affect brain function. Therefore, unique brain mechanisms of itch may be involved in atopic dermatitis; however, its molecular basis remains largely unclear.

We previously reported a unique, diet-induced chronic mouse model of atopic dermatitis. HR-1 hairless mice fed a special diet (named HR-AD) develop atopic dermatitis-like skin inflammation^7,8^. Interestingly, in this model, administration of certain central nervous system (CNS) drugs such as ethanol and barbiturates, markedly increased scratching^9,10^. Barbiturate-induced scratching was replicated in another chronic dermatitis model NC/Nga mice but not in the histamine-induced acute itch model using normal healthy mice^10^, suggesting that such enhancement of scratching is characteristic of chronic disease conditions. Further, we have shown that the CNS drug-induced scratching could be attributed, at least partly, to a synergistic effects on multiple targets including γ-aminobutyric acid type A (GABA_A_) receptors, *N*-methyl-D-aspartate (NMDA) and α-amino-3-hydroxy-5-methyl-4-isoxazolepropionic acid (AMPA) glutamate receptors, and L-type voltage-dependent calcium channels (L-VDCC)^9,10^; however, whether endogenous substances acting on these targets contribute to pruritus remains to be studied.

3α,5α-Tetrahydroprogesterone, also called allopregnanolone (ALLO), is one of the neurosteroids that are synthesized de novo in the brain or reach the brain from peripheral steroidogenic organs, such as adrenals and gonads^11^. Neurosteroids not only act on gene expression via intracellular steroid hormone receptors, but also rapidly alter neuronal excitability by acting through various membrane receptors^12,13^. Specifically, ALLO has been shown to positively modulate GABA_A_ receptors, thereby producing barbiturate-like neurobehavioral effects such as anxiolytic, anticonvulsant, and sedative/hypnotic actions^14,15^. It has also been reported that similar to ethanol and barbiturates, ALLO inhibits L-VDCC^16^. Since ALLO has some similar pharmacological properties to those of ethanol and barbiturates, we hypothesized that it is involved in pruritus in atopic dermatitis.

In the present study, to prove this hypothesis, we first investigated whether ALLO administration causes scratching in the diet-induced atopic dermatitis model. Second, we also determined whether endogenously produced ALLO is involved in ethanol-induced scratching in the same model, because acute ethanol administration has been shown to increase brain ALLO levels in rodents^17–19^.

## Results

### Systemic administration of ALLO elicits robust scratching in special diet-fed atopic dermatitis model, while it did not affect spontaneous and pruritogen-induced scratching in normal mice

**Figure 1:**
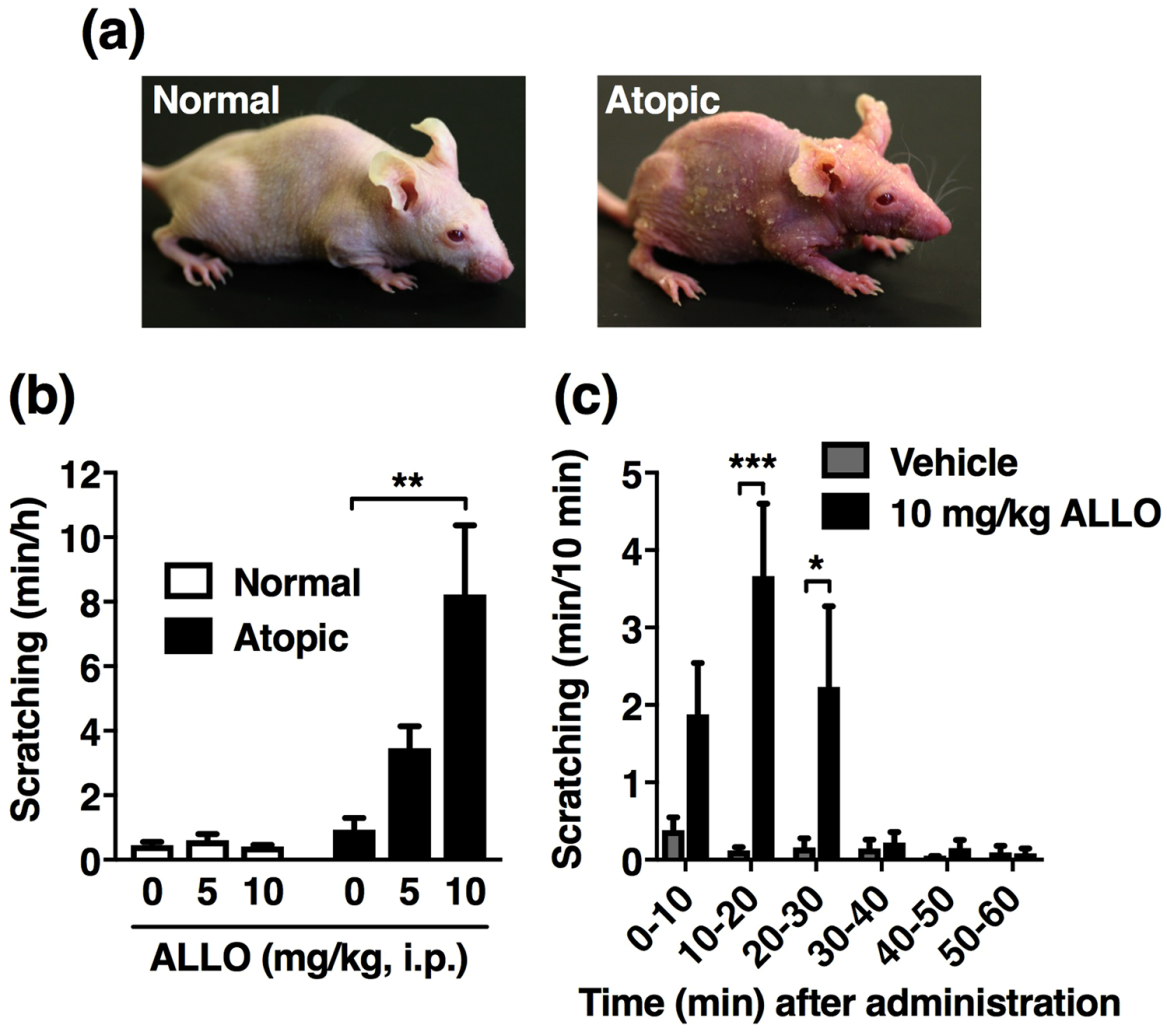
Induction of scratching behaviour by intraperitoneal (i.p.) administration of allopregnanolone (ALLO) to atopic dermatitis-induced mice. (**a**) Mouse fed normal diet (left panel, Normal) or special diet (right panel, Atopic) for 12 weeks. (**b**) Vehicle (20% castor oil in saline) or ALLO (5 and 10 mg/kg) was i.p. administered to normal and atopic dermatitis-induced mice, and cumulative duration of scratching was measured for 1 h. Each column represents mean ± S.E.M. of six animals. **P < 0.01, vs. vehicle (designated as 0 mg/kg), one-way ANOVA with Dunnett’s multiple comparison test. (**c**) Time course of the cumulative duration of scratching when vehicle or 10 mg/kg ALLO was administered to atopic dermatitis-induced mice. Each column represents the mean ± S.E.M. of six animals. *P < 0.05 and ***P < 0.001, vs. vehicle, two-way ANOVA with Bonferroni’s multiple comparison test.

Consistent with our previous results^7,8^, special diet-fed hairless mice exhibited red scaly skin resembling that observed in human atopic dermatitis (Fig. 1a, right panel), whereas normal diet-fed mice had no gross abnormalities (Fig. 1a, left panel). When ALLO (5 and 10 mg/kg) was administered intraperitoneally (i.p.) to normal mice, no significant change occurred in the cumulative duration of hindlimb scratching (Fig. 1b). In contrast, in the special diet-fed atopic dermatitis mouse model, the scratching duration was dose-dependently increased by ALLO administration, with a significant effect observed at 10 mg/kg (Fig. 1b). The mice administered ALLO frequently scratched their face and ears (Supplemental video-1), although sometimes also their back and trunk. The time course of scratching showed that ALLO-induced scratching started within 10 min after administration, peaked at 10–20 min, and had almost subsided at 30 min (Fig. 1c); thus, this scratching response was probably mediated by a non-genomic action.

**Figure 2:**
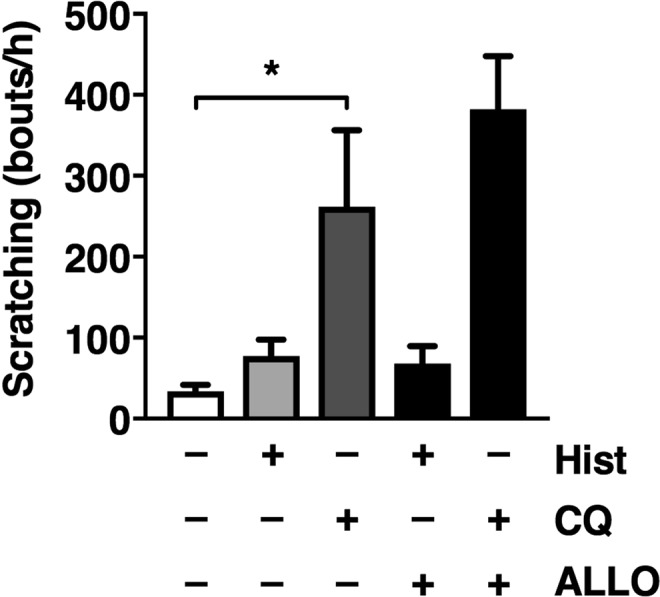
No effect of allopregnanolone (ALLO) on pruritogen-induced scratching in normal mice. Vehicle (saline) or ALLO (10 mg/kg, i.p.) was administered to normal mice, and 10 min later, saline (50 µL/site), histamine (Hist, 250 µg/50 µL/site) or chloroquine (CQ, 200 µg/50 µL/site) was i.d. injected to the nape of the neck. Immediately after i.d. injection, the number of scratching bouts on the injection site was measured for 1 h. Each column represents the mean ± S.E.M. of four animals. *P < 0.05, one-way ANOVA with Bonferroni’s multiple comparison test.

We next examined whether ALLO administration also increases pruritogen-induced acute scratching in normal mice. However, systemic administration of ALLO (10 mg/kg, i.p.) did not significantly affect histamine- or chloroquine-induced scratching (Fig. 2).

### ALLO acts supraspinally to induce scratching

**Figure 3:**
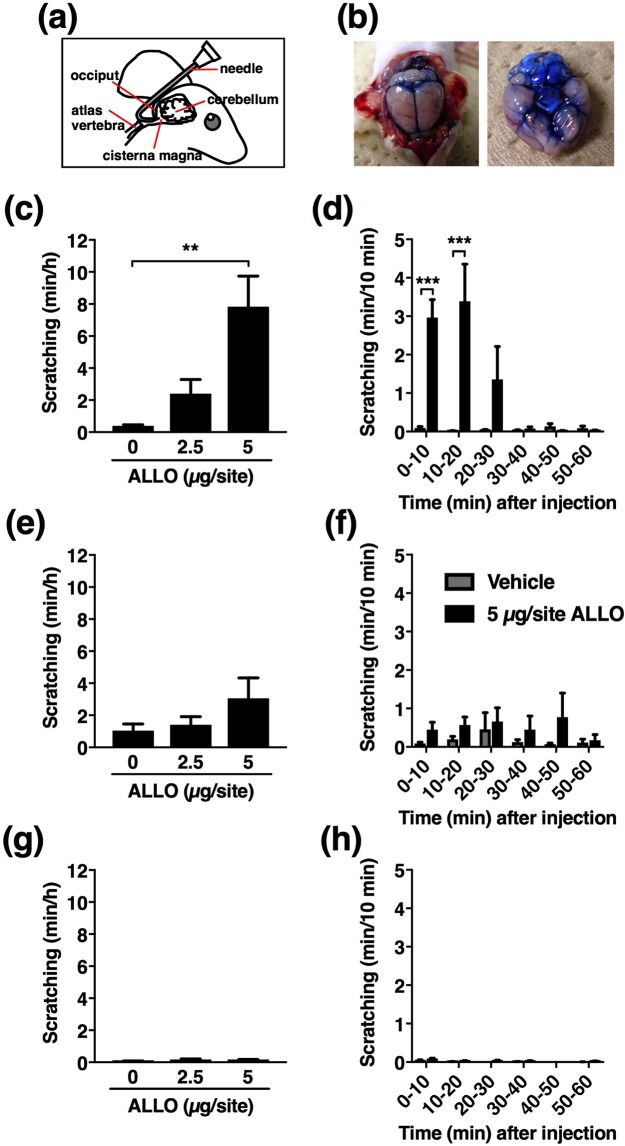
Induction of scratching behaviour by an intracisternal (i.ci.) injection of low-dose allopregnanolone (ALLO) to atopic dermatitis-induced mice. (**a**) Schematic illustration of i.ci. injection. (**b**) Gross distribution of 1% Evans blue solution i.ci. injected at 10 µL. (**c**,**e**,**g**) Vehicle (20% castor oil in saline, 10 µL) or ALLO (2.5 and 5 µg/10 µL in each site) was i.ci. (**c**), intrathecally (i.t.) (**e**), or intradermally (i.d.) (**g**) injected into atopic dermatitis-induced mice, and from immediately (for i.ci. and i.d.) or 5 min (for i.t.) after injection, the cumulative duration of scratching was measured for 1 h. Each column represents the mean ± S.E.M. of five animals. **P < 0.01, vs. vehicle (designated as 0 µg/site), one-way ANOVA with Dunnett’s multiple comparison test. (**d**,**e**,**h**) Time course of the cumulative duration of scratching when vehicle or 5 µg/site ALLO was i.ci. (**d**), i.t. (**f**), or i.d. (**h**) administered to atopic dermatitis-induced mice. Each column represents the mean ± S.E.M. of five animals. ***P < 0.001, vs. vehicle, two-way ANOVA with Bonferroni’s multiple comparison test.

To determine the site of action of ALLO-induced scratching, a low dose of ALLO was locally (intracisternal [i.ci.], intrathecal [i.t.], or intradermal [i.d.]) injected to the atopic dermatitis-induced mice. The i.ci. injection of ALLO (2.5 and 5 µg/site) dose-dependently and significantly induced scratching (Fig. 3c), which was comparable to that induced by i.p. administration (Fig. 1b). Time course and manner of scratching after i.ci. injection of ALLO (Fig. 3d and Supplemental video-2) was very similar to that of i.p. administration (Fig. 1c and Supplemental video-1). On the other hand, i.t. and i.d. injections at the same dose did not cause significant scratching (Fig. 3e–h).

### ALLO-induced scratching is suppressed by GABA_A_ receptor antagonist and serotonin 5-HT_3_ receptor agonist

**Figure 4:**
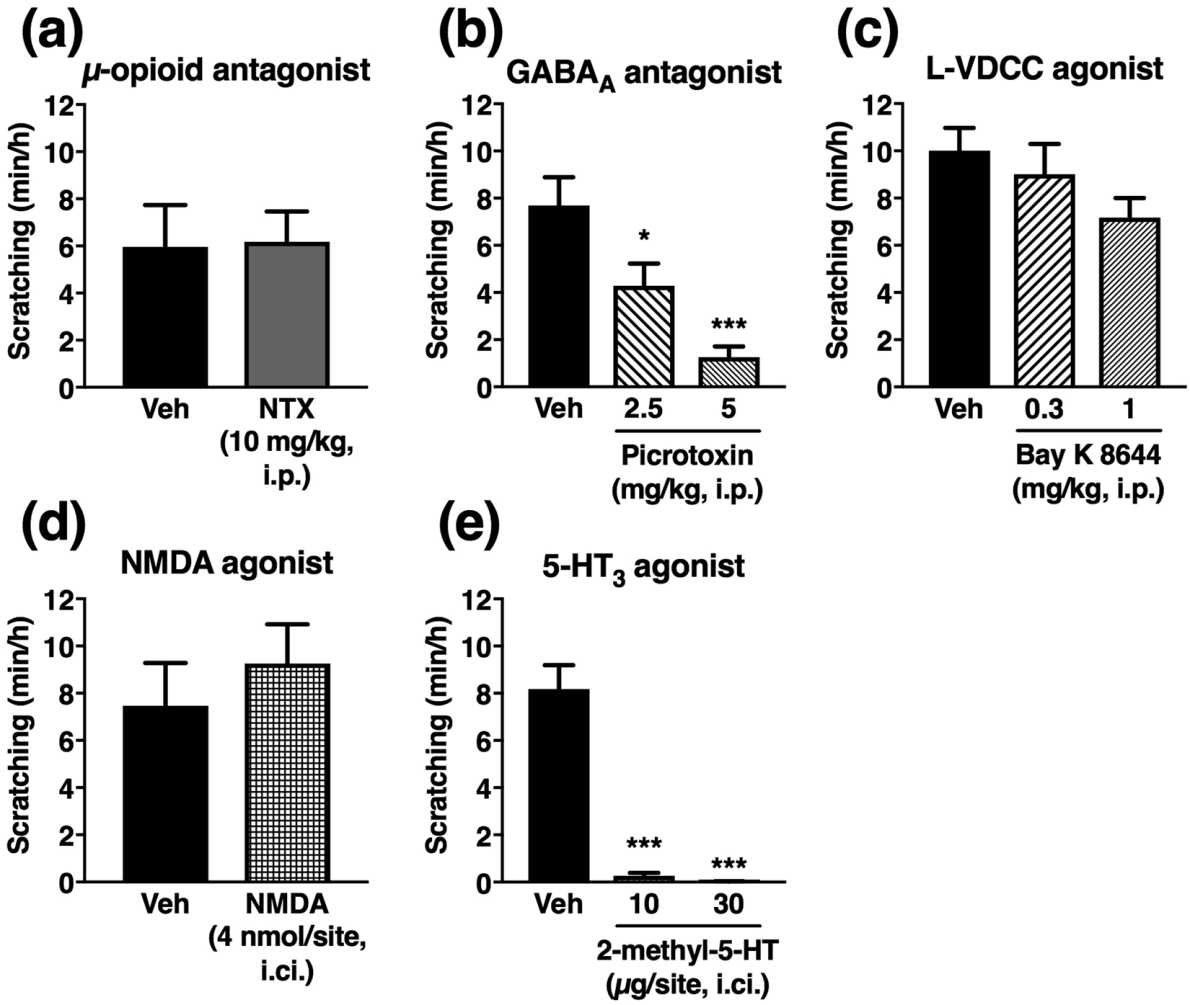
Effects of several drugs on allopregnanolone (ALLO)-induced scratching in atopic dermatitis-induced mice. ALLO was i.p. administered at 10 mg/kg. (**a**) Vehicle (saline) or naltrexone (NTX, 10 mg/kg) was i.p. administered 30 min before ALLO administration. (**b**) Vehicle (saline) or picrotoxin (2.5 or 5 mg/kg) was i.p. administered 5 min after ALLO administration. (**c**) Vehicle (0.5% Tween 80-containing saline) or Bay K 8644 (0.3 or 1 mg/kg) was i.p. administered 5 min after ALLO administration. (**d**) Vehicle (saline) or NMDA (4 nmol/10 µL/site) was i.ci. administered 5 min after ALLO administration. (**e**) Vehicle (saline) or 2-methyl-5-HT (10 or 30 µg/10 µL per site) was i.ci. administered 5 min after ALLO administration. Each column represents the mean ± S.E.M. of six (**a**), eight or nine (b), 16 or 17 (**c**), six or seven (**d**), and five to six (**e**) animals. *P < 0.05 and ***P < 0.001, vs. vehicle (Veh), unpaired Student’s *t-*test or one-way ANOVA with Dunnett’s multiple comparison test.

Next, we pharmacologically analysed the mechanism underlying ALLO-induced scratching. Opioid receptor antagonists, such as naloxone and naltrexone, have been demonstrated to suppress various types of pruritus^20–22^. Thus, we first examined the effect of naltrexone on ALLO-induced scratching. However, naltrexone, even at the high dose (10 mg/kg, i.p.), did not suppress scratching (Fig. 4a), suggesting that ALLO-induced scratching is not a common opioid-mediated itch response.

We next examined whether activation of GABA_A_ receptors is required for ALLO-induced scratching, because ALLO is a potent, positive allosteric modulator of GABA_A_ receptors^14,15^. When the GABA_A_ receptor antagonist picrotoxin was administered i.p. 5 min after i.p. administration of ALLO, the scratching response was significantly suppressed depending on the dose of picrotoxin (Fig. 4b). This result supports the hypothesized role of GABA_A_ receptors in ALLO-induced scratching, but we postulated the involvement of other mechanisms because we previously found that the selective GABA_A_ receptor agonist muscimol failed to induce scratching in the same model^10^.

Similar to ethanol and barbiturates, ALLO inhibits the L-VDCC^16^, and our previous^10^ and unpublished results showed that both ethanol- and barbiturate-induced scratching in atopic dermatitis mice were significantly suppressed by co-administration of the L-VDCC agonist BAY K 8644 (0.3 and 1 mg/kg, i.p.). Thus, we evaluated the effect of the same dose of BAY K 8644 on ALLO-induced scratching, and found that BAY K 8644 tended to suppress ALLO-induced scratching, but the effect was not statistically significant (Fig. 4c).

Although there is no convincing evidence that ALLO acts on glutamate receptors, we previously found that an i.ci. injection of NMDA (4 nmol/site) significantly blocked ethanol-induced scratching in atopic dermatitis in mice^9^. Accordingly, we examined the effects of NMDA at the same dose on ALLO-induced scratching, but it did not suppress scratching (Fig. 4d).

Wetzel *et al*.^23^ have demonstrated that ALLO acts as a functional antagonist of serotonin 5-hydroxytryptamine type 3 (5-HT_3_) receptors. Thus, we examined the effect of the 5-HT_3_ receptor agonist 2-methyl-5-HT on ALLO-induced scratching. When 2-methyl-5-HT was i.ci. injected 5 min after ALLO administration, scratching behaviour was almost completely suppressed (Fig. 4e). On the other hand, the i.ci. injection of 2-methyl-5-HT, even at the high dose (30 µg/site), did not significantly affect locomotor activity (data not shown). This observation suggested that suppression of ALLO-induced scratching by 2-methyl-5-HT was not a simple side-effect of the drug.

### Concomitant increase in scratching behaviour and brain ALLO levels after acute ethanol administration

**Figure 5:**
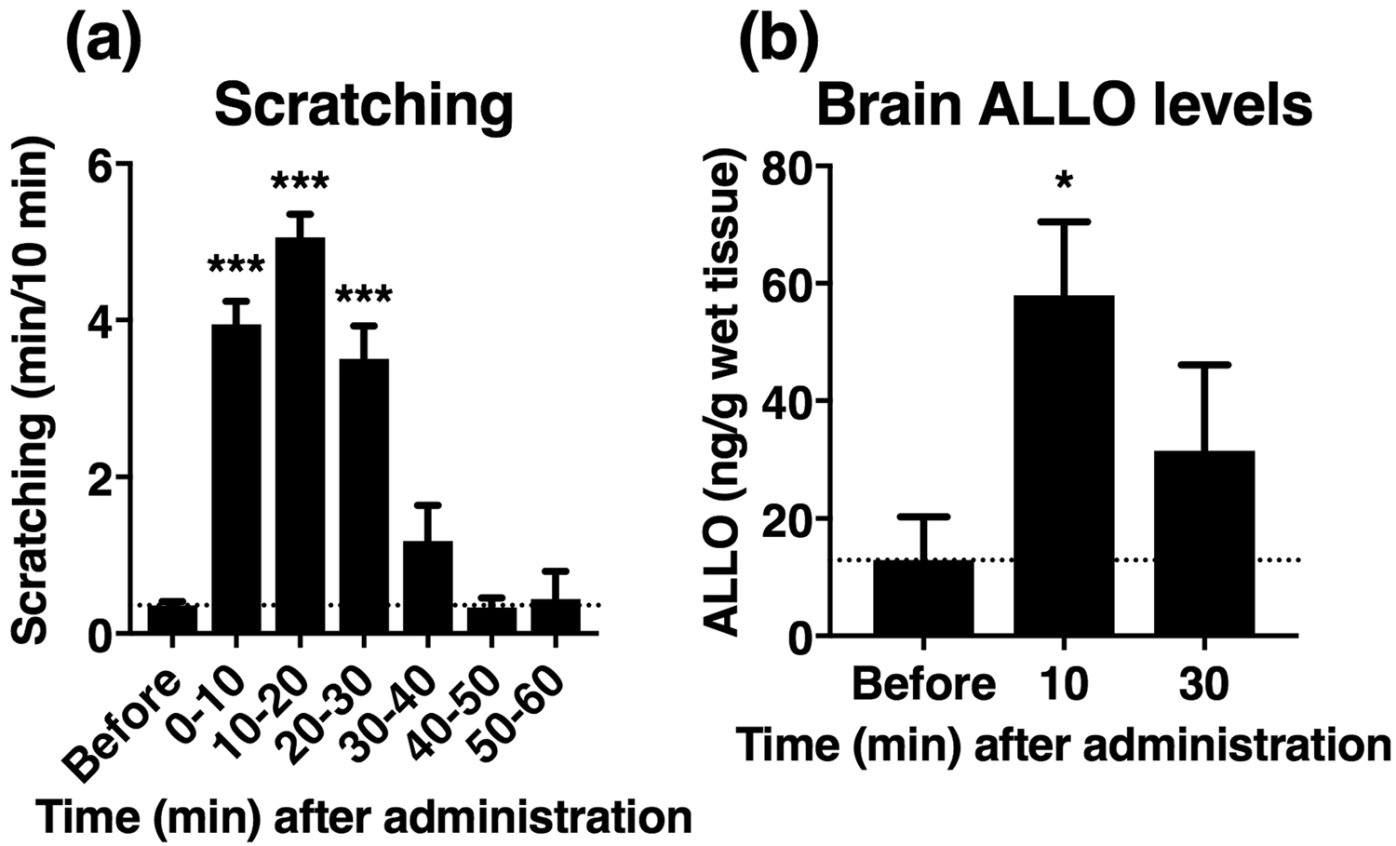
Time course of scratching and brain allopregnanolone (ALLO) levels following ethanol administration. (**a**,**b**) Ethanol (2.4 g/kg) was p.o. administered to atopic dermatitis-induced mice and the cumulative duration of scratching (**a**) and the brain ALLO levels (**b**) were measured at indicated time points. Each column represented means ± S.E.M. of nine or 11 (**a**) and six or seven (**b**) animals. *P < 0.05 and ***P < 0.001, vs. before, one-way ANOVA with Dunnett’s multiple comparison test.

It has been shown that acute administration of ethanol (1.35–4 g/kg) rapidly increases brain ALLO levels in rodents^17–19^. Further, we previously showed that oral administration of ethanol (2.4 g/kg) markedly increased scratching in atopic dermatitis in mice^9^. Based on these previous findings and the present results, we hypothesized that ALLO endogenously produced in the brain contributes to the ethanol-induced scratching. To test this hypothesis, we investigated the time-course of scratching behaviour and brain ALLO levels after administering ethanol (2.4 g/kg, orally [p.o.]) to the atopic dermatitis mouse model. Consistent with previous results^9^, the scratching behaviour was apparently increased for 0–30 min after ethanol administration (Fig. 5a). The brain ALLO levels were also significantly elevated at 10 min and remained high for up to 30 min after ethanol administration, compared with the basal levels (Fig. 5b).

### Pre-treatment with finasteride suppresses ethanol-induced scratching and brain ALLO production

**Figure 6:**
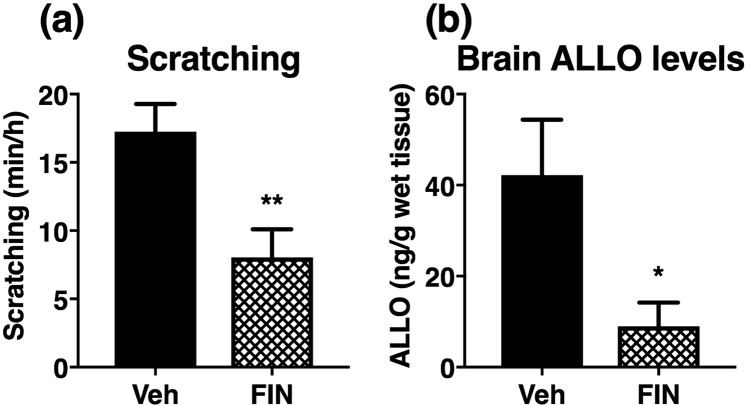
Suppression of ethanol-induced scratching and brain allopregnanolone (ALLO) production in atopic dermatitis-induced mice pre-treated with the 5α-reductase inhibitor, finasteride. (**a**,**b**) Vehicle (20% β-cyclodextrin-containing saline) or finasteride (FIN, 50 mg/kg, i.p.) was twice administered 24 h and 1 h before administration of ethanol (2.4 g/kg, p.o.). (**a**) Cumulative duration of scratching during 1 h period after ethanol administration. (**b**) Brain ALLO levels 10 min after ethanol administration. Each column represents mean ± S.E.M. of (**a**) 16 or 17 and (**b**) four or five animals. *P < 0.05 and **P < 0.01, vs. vehicle (Veh), unpaired Student’s *t-*test.

Finally, we investigated whether the ethanol-induced increase in brain ALLO levels contributes to scratching. ALLO has been shown to be synthesized from progesterone via 5α-reductase type 1, which is the rate-limiting enzyme in ALLO production^11^. Thus, we examined the effects of the 5α-reductase inhibitor finasteride on the scratching response during 1 h and the brain ALLO levels at 10 min after ethanol administration. As a result, pre-treatment with finasteride significantly suppressed ethanol-induced scratching and ALLO production in the brain (Fig. 6).

## Discussion

This study examined whether endogenous neurosteroid ALLO is involved in pruritus in an atopic dermatitis mouse model. We found that systemic administration of ALLO markedly increased spontaneous scratching in mice with atopic dermatitis, while it did not affect spontaneous and pruritogen-induced scratching in normal mice. Local injection studies showed that ALLO acted at the supraspinal level to cause scratching. Thus, our data showed that the brain ALLO content may contribute to inducing scratching in atopic dermatitis. Although ALLO is the best characterized neurosteroid and has been extensively studied in various CNS disorders^24^, to the best of our knowledge, this is the first study to show the possible link between brain ALLO and itch-like behaviour.

Because animals cannot describe their sensations, it is difficult to conclude that ALLO-induced hindlimb scratching is really caused by increased itch sensation; however, numerous studies support the idea that hindlimb scratching in mice essentially reflects itch sensation^25,26^. µ-Opioid antagonists relieve various types of pruritus and, thus, are useful to evaluate whether animal scratching is an itch-related behaviour; however, they are not always effective in human and animal itch cases^27–29^. In our atopic dermatitis model, although neither the cumulative duration nor the frequency of spontaneous scratching bouts constantly increased, the duration of one scratching bout was reproducibly prolonged, and was suppressed by naloxone^21^. On the other hand, ethanol- and barbiturate-induced scratching were partially and not significantly suppressed, respectively by naltrexone at the same dose used in the present study^9,10^. We presently found that ALLO-induced scratching was insensitive to naltrexone treatment. Therefore, we assumed that the marked scratching response induced by ethanol, barbiturates, or ALLO was essentially different from common opioid-mediated itch responses. Rather, since similar to ethanol and barbiturates, ALLO exerts a hypnotic effect^30,31^, the unique scratching phenomenon might resemble the unconscious nocturnal pruritus frequently observed in patients with atopic dermatitis^32–34^.

ALLO has been shown to act as a potent GABA_A_ receptor agonist^15^ and circulating ALLO reached brain tissues, which correlated with its CNS effects^31,35^. In this study, we found that ALLO-induced scratching was completely blocked by an i.p. administration of the GABA_A_ receptor antagonist picrotoxin. It is widely accepted that systemically administered picrotoxin primarily acts on GABA_A_ receptors in the brain. Furthermore, our preliminary experiment showed that i.ci. administered picrotoxin similarly suppressed i.p. ALLO-induced scratching in another but similar diet-induced atopic dermatitis mouse model (data not shown). Therefore, ALLO-induced scratching could be attributed to activation of brain GABA_A_ receptors. We previously showed that other GABAergic drugs such as ethanol and barbiturates also markedly enhanced scratching in atopic dermatitis in mice, which was suppressed by GABA_A_ receptor antagonists^9,10^. On the other hand, benzodiazepines only slightly enhanced scratching, whereas the selective GABA_A_ receptor agonist muscimol decreased scratching^10^. The discrepancy between the effects of these GABAergic substances on scratching has two possible explanations. The first is the possible involvement of additional targets other than GABA_A_ receptors. Although benzodiazepines and muscimol act exclusively on GABA_A_ receptors, ethanol, barbiturates, and ALLO have many additional targets^13,36,37^. Indeed, our previous results suggest that inhibition of NMDA glutamate receptors and L-VDCC may contribute to ethanol-induced scratching in atopic dermatitis in mice^9^, and that inhibition of AMPA glutamate receptors and L-VDCC could be involved in barbiturate-induced scratching^10^. On the other hand, we showed that co-application of the 5-HT_3_ receptor agonist almost completely blocked ALLO-induced scratching, although either an L-VDCC agonist or NMDA had no significant effect. The sum of the inhibitory rates of ALLO-induced scratching induced by the GABA_A_ receptor antagonist and 5-HT_3_ receptor agonist was much greater than 100%. Therefore, some synergistic interaction between GABA_A_ receptor activation and 5-HT_3_ receptor inhibition in the brain may be required for ALLO-induced scratching. It should also be noted that the scratching responses caused by ethanol, barbiturates, and ALLO have distinctively different underlying mechanisms other than activation of GABA_A_ receptors.

Second, GABA_A_ receptor heterogeneity may also be involved in ALLO-induced scratching. There are various subtypes of GABA_A_ receptors with distinct functions and pharmacology^38^, but they can be divided into two subtypes based on the presence of γ or δ subunits. The γ- and δ-containing receptors are mainly located synaptically and extrasynaptically, respectively, and thereby contribute to phasic and tonic GABA-mediated inhibitory transmission, respectively^39^. Although benzodiazepines specifically bind to γ-containing receptors, ALLO, ethanol, and barbiturates act on both receptor subtypes^37,39–41^. Importantly, although muscimol is generally regarded as a GABA_A_ receptor pan-agonist, its *in vivo* effects are reported to be mediated preferentially through a small population of δ-containing receptors^42,43^. Considering these between-drug differences in the effects on GABA_A_ receptors, activation of both GABA_A_ receptor subtypes may also be necessary to fully induce scratching in atopic dermatitis mice. However, further studies using γ or δ subunit knockout mice are warranted to test this hypothesis.

The present study suggested that both GABA_A_ receptor activation and 5-HT_3_ receptor inhibition contributed to ALLO-induced scratching. This conclusion is highly consistent with pharmacological studies on ALLO^12^, but seems inconsistent with reports on the role of these receptors in certain itch conditions. Several studies have shown that GABA_A_ receptors in the spinal cord and central nucleus of the amygdala have an inhibitory role in itch signalling and the resultant scratching behaviour^44–46^. Moreover, activation of 5-HT_3_ receptors has been reported to contribute to certain types of pruritus^47–50^. However, this discrepancy could be explained by the following differences between these previous results and our present findings. (1) ALLO-induced scratching was not mediated through spinal GABA_A_ receptors, although the effects on the receptors in the central nucleus of the amygdala could not be excluded. (2) In the previous studies, muscimol was used as an agonist for GABA_A_ receptors to inhibit scratching responses^45,46^. On the other hand, ALLO caused scratching probably by pharmacological properties that differed from those of muscimol (as described above), whereas muscimol similarly reduced scratching in our model^10^. (3) Although 5-HT_3_ antagonists are highly effective against opioid-induced pruritus in humans^47,48^, ALLO-induced scratching is intrinsically independent on opioid receptors. (4) Activation of 5-HT_3_ receptors in the skin or spinal cord is considered to contribute to induction of scratching in pruritogen-induced acute and cholestatic itch models^49,50^. On the other hand, in our atopic dermatitis model, ALLO inhibited this receptor at the supraspinal level, and eventually likely contributed to the induction of scratching. (5) Inhibition of scratching by muscimol was observed in both acute and chronic itch conditions^45,46^, whereas ALLO-induced scratching was observed only in chronic dermatitis in mice. Therefore, considering these differences, it is reasonable that ALLO could induce scratching in certain chronic dermatitis conditions through an unknown mechanism.

In the present study, we found a concomitant increase in scratching behaviour and brain ALLO levels after acute ethanol administration, which was suppressed by pre-treatment with the ALLO synthetic inhibitor finasteride. Furthermore, our preliminary experiment showed that the increased levels of ALLO after ethanol administration were comparable to that after an i.p. administration of 10 mg/kg ALLO (data not shown). These results support our hypothesis that endogenously produced ALLO partly contributed to ethanol-induced scratching in the atopic dermatitis mouse model. It has been reported that the two enzymes responsible for ALLO synthesis (i.e., 5α-reductase type I and 3α-hydroxysteroid dehydrogenase) are colocalized in the cortical, hippocampal, and olfactory bulb glutamatergic principal neurons and in some output neurons of the amygdala and thalamus^51^. However, the brain region contributing to scratching induction remains to be determined and needs further investigation. Furthermore, a number of studies have shown that the expression levels of these enzymes and ALLO concentrations are altered under various physiological and disease conditions^14^. Therefore, it would be interesting to determine whether fluctuation of ALLO in some specific brain regions contributes to scratching behaviour in various physiological situations such as sleep, stress, and anxiety, which are all known to often exacerbate pruritus in pathological conditions^6,52^.

ALLO-induced enhancement of scratching was observed only in chronic atopic dermatitis mice, which is consistent with our previous findings using ethanol^9^ or barbiturates^10^. Therefore, such enhancement of scratching may be associated with highly chronic disease conditions. The present study showing that the same dose of ALLO induced scratching in atopic but not normal mice suggests that this difference is attributed likely to some differences of responsivity to ALLO (e.g., the expression or function of the related receptors, or the existence of unique nervous system under chronic disease conditions) only rather than the brain content of ALLO.

In conclusion, this study demonstrated that brain ALLO induced marked scratching in atopic dermatitis in mice, and suggested that ethanol-induced scratching is mediated partly through endogenously produced ALLO in the brain. Therefore, brain ALLO may be involved in pruritus in atopic dermatitis. However, as yet there is no direct evidence that endogenous brain ALLO contributes to naturally occurring itch, we need further studies using another chronic itch model that shows spontaneous scratching. In addition to ALLO, a variety of neurosteroids are present in the brain such as tetrahydro-deoxycorticosterone and pregnenolone sulphate, which exert similar and opposite actions to ALLO, respectively^12^. Further investigation of the roles of brain neurosteroids in pruritus may enhance the understanding of supraspinal mechanisms of chronic itch and could lead to the development of novel therapeutic approaches to intractable pruritus in chronic diseases including atopic dermatitis.

## Methods

### Animals

Four-week-old, female hairless mice (Hos: HR-1) were purchased from Hoshino Laboratory Animals (Saitama, Japan) and were maintained in plastic cages with free access to food and water and housed at 22 ± 1 °C on a 12-h light/dark cycle. All animal studies were approved by the Ethics Committee of Animal Research of Kyoto Pharmaceutical University (Approval numbers: 16–12–054 and 17–035), and were performed in accordance with the Guidelines for Proper Conduct of Animal Experiments (Science Council of Japan, 2006).

### Diet-induced atopic dermatitis model

As reported previously^7^, hairless mice were fed a special diet (HR-AD diet; Norsan, Yokohama, Japan) for 8–12 weeks to fully induce atopic dermatitis-like symptoms and then they were used within 1 week. The disease signs were confirmed by gross observation, measurement of skin hydration, and transepidermal water loss using a Corneometer® CM825 and Tewameter® TM210 (both from Courage + Khazaka Electronic, Cologne, Germany), respectively. Mice fed a standard laboratory diet (MF; Oriental Yeast Industry, Tokyo, Japan) were used as the negative control.

### Drug preparation

The reagents were prepared as follows: ALLO (Sigma-Aldrich, St. Louis, MO, USA) was dissolved in a mixture of castor oil (Cremophor^®^ EL; Sigma-Aldrich) and physiological saline (1:4, v/v). Chloroquine diphosphate, histamine dihydrochloride, naloxone hydrochloride, picrotoxin, NMDA (all from Sigma-Aldrich), and 2-methyl-5-HT (both from Wako Pure Chemical Industries, Osaka, Japan) were dissolved in physiological saline. (−)-BAY K 8644 (Sigma-Aldrich) was suspended in physiological saline containing 0.5% (v/v) Tween 80. Ethanol (Wako Pure Chemical Industries) was diluted in purified water. Finasteride (Cayman Chemical, Ann Arbor, MI, USA) was dissolved in 20% (w/v) β-cyclodextrin-containing physiological saline. Each drug was prepared immediately before use.

### Drug administration

The i.ci. injection was performed in conscious animals, as described by Ueda *et al*.^53^ with slight modifications. Briefly, the head of each mouse was gently bent and a J-shaped needle (the 27-gauge stainless needle was curved 40 degrees at 3.5 mm from the tip) was inserted into the cleft between the occiput (Fig. 3a). Then, 10 µL aqueous solution was gradually injected into the cisternal magna. Using 1% Evans blue solution, we confirmed that the solution was primarily distributed in the area surrounding the cisternal magna and the ventral surface of the brain stem (Fig. 3b). The i.t. injection was performed under brief isoflurane (2%) anaesthesia, induced with 10 µL injected via lumbar puncture according to a modified method^54^. For the i.d. injection, 10 µL of the solution was injected into the nape of the neck using a 29-gauge butterfly needle, while p.o. administration was performed using a gavage needle (#5202; Fuchigami Kikai, Kyoto, Japan).

### Analysis of scratching behaviour

Hindlimb scratching in mice is thought to be associated with itch sensation^25,26^. In the present study, all hindlimb scratching behaviours were analysed regardless of scratching sites, except for the i.d. injection, and only scratching directed to the area around the injection site was analysed.

Prior to observing scratching, the mice were acclimatized for a minimum of 10 min in an observation chamber. Immediately after drug administration, the scratching behaviour was recorded and then analysed by playing back the videotape as reported previously^7^. Briefly, the cumulative duration of scratching was determined by instructing the observer to touch the switch for the duration of the scratching behaviour, using an in-house counter. The time detectable by this instrument was 0.1 s. Exceptionally, pruritogen (histamine or chloroquine)-induced scratching response was evaluated by measuring the number of scratching bouts on the injected site.

### Determination of brain ALLO levels

Immediately after euthanasia by cervical dislocation, whole brain samples were collected and stored at −80 °C until extraction. The obtained brain samples were homogenized in 10 volumes of ethyl acetate, the supernatants were evaporated under nitrogen stream, and then stored at −80 °C until the analysis. The amount of ALLO was determined using a commercially available enzyme-linked immune assay kit (ELISA, DirectX^®^ Allopregnanolone immunoassay kit; Arbor Assays, Ann Arbor, MI, USA) according to the manufacture’s instructions. The data were normalized to the wet tissue weight.

### Experimental procedures

In the experiment examining the effect of systemic administration of ALLO on spontaneous scratching, ALLO (5 and 10 mg/kg) was administered i.p. to normal mice and the atopic dermatitis model mice and then the scratching behaviour was recorded for 1 h. Histamine (250 µg/50 µL/site) or chloroquine (200 µg/50 µL/site) was i.d. injected 10 min after administration of ALLO (10 mg/kg, i.p.) and then scratching behaviour was recorded for 1 h. In the experiment examining the effects of local injection of ALLO on scratching, low doses of ALLO (2.5 and 5 µg/10 µL/site) were injected i.ci., i.t., or i.d. into atopic dermatitis-induced mice, and from immediately (for i.ci. and i.d.) or 5 min (for i.t. to recover from isoflurane anesthesia) after injection, the scratching behaviour was recorded for 1 h. For the co-administration with ALLO (10 mg/kg, i.p.) in atopic dermatitis-induced mice, the dose, route, and timing of administration were as follows. Naltrexone (10 mg/kg) was i.p. administered 30 min before ALLO administration. Picrotoxin (2.5 and 5 mg/kg) or BAY K 8644 (0.3 and 1 mg/kg) was i.p. administered 5 min after ALLO administration. NMDA (4 nmol/site) or 2-methyl-5-HT (10 and 30 µg/site) was i.ci. injected 5 min after ALLO administration, because these drugs cannot possibly pass through the blood-brain barrier. In these experiments, scratching behaviour was recorded for 1 h after administration of the latter drug. For determining the time course of scratching and the brain ALLO levels after ethanol administration, ethanol (2.4 g/kg) was p.o. administered to atopic dermatitis mice, and then the scratching behaviour was recorded for 1 h. Furthermore, the brain samples of another group of mice were collected at 10 and 30 min after ethanol administration. In the experiment examining the effects of finasteride on ethanol-induced scratching and brain ALLO production in atopic dermatitis mice, finasteride (50 mg/kg) was i.p. administered twice at 24 and 1 h before ethanol administration (2.4 g/kg, p.o.). Following the ethanol administration, scratching behaviour was recorded for 1 h and in another sets of mice the brain samples were collected at 10 min after ethanol administration. The control group received the vehicle of each drug. All selected doses were based on the results of our previous^9,10^, preliminary *in vivo* experiments, and those of *in vitro* and *in vivo* experiments reported by others^16,23,55^; the doses of ALLO by systemic (5 and 10 mg/kg, i.p.) or local (2.5 and 5 µg/site, i.ci.) administration have shown to produce antiepileptic or sedative/hypnotic effects in mice and rats^56–58^.

### Statistical analysis

Data were analysed using the GraphPad Prism (version 7.0; GraphPad Software, San Diego, CA, USA) and are presented as the means ± standard error of the mean (S.E.M.). Statistical differences were determined using unpaired Student’s *t-*test, a one-way analysis of variance (ANOVA) with Dunnet’s multiple comparison test or Bonferroni’s multiple comparison test, or two-way ANOVA with Bonferroni’s multiple comparison test. Differences were considered significant at *P* < 0.05.

## Supplementary information

Supplemental video-1

Supplemental video-2

Supplemental video legends

## References

[1] Kido-Nakahara M, Furue M, Ulzii D, Nakahara T (2017). Itch in AtopicDermatitis. *Immunol*. Allergy Clin. North Am..

[2] Nakashima C, Otsuka A, Kabashima K (2018). Interleukin-31 and interleukin-31 receptor: New therapeutic targets for atopic dermatitis. Exp. Dermatol..

[3] Rerknimitr P, Otsuka A, Nakashima C, Kabashima K (2017). The etiopathogenesis of atopic dermatitis: barrier disruption, immunological derangement, and pruritus. Inflamm. Regen..

[4] Tsuda M (2018). Astrocytes in the spinal dorsal horn and chronic itch. Neurosci. Res..

[5] Werfel T, Biedermann T (2015). Current novel approaches in systemic therapy of atopic dermatitis: specific inhibition of cutaneous Th2 polarized inflammation and itch. Curr. Opin. Allergy Clin. Immunol..

[6] Ständer S, Steinhoff M (2002). Pathophysiology of pruritus in atopic dermatitis: an overview. Exp. Dermatol..

[7] Fujii M (2005). Atopic dermatitis-like pruritic skin inflammation caused by feeding a special diet to HR-1 hairless mice. Exp. Dermatol..

[8] Fujii M (2015). Dietary deficiencies of unsaturated fatty acids and starch cause atopic dermatitis-like pruritus in hairless mice. Exp. Dermatol..

[9] Fujii M (2009). Ethanol aggravates itch-related scratching in hairless mice developing atopic dermatitis. Eur. J. Pharmacol..

[10] Fujii M (2018). Barbiturates enhance itch-associated scratching in atopic dermatitis mice: a possible clue to understanding nocturnal pruritus in atopic dermatitis. Eur. J. Pharmacol..

[11] Baulieu EE (1991). Neurosteroids: a new function in the brain. Biol. Cell..

[12] Dubrovsky BO (2005). Steroids, neuroactive steroids and neurosteroids in psychopathology. Prog. Neuropsychopharmacol. Biol. Psychiatry..

[13] Rupprecht R, Holsboer F (1999). Neuroactive steroids: mechanisms of action and neuropsychopharmacological perspectives. Trends Neurosci..

[14] Belelli D, Lambert JJ (2005). Neurosteroids: endogenous regulators of the GABA(A) receptor. Nat. Rev. Neurosci..

[15] Majewska MD, Harrison NL, Schwartz RD, Barker JL, Paul SM (1986). Steroid hormone metabolites are barbiturate-like modulators of the GABA receptor. Science..

[16] Earl DE, Tietz EI (2011). Inhibition of recombinant L-type voltage-gated calcium channels by positive allosteric modulators of GABAA receptors. J. Pharmacol. Exp. Ther..

[17] Gabriel KI, Cunningham CL, Finn DA (2004). Allopregnanolone does not influence ethanol-induced conditioned place preference in DBA/2J mice. Psychopharmacology (Berl)..

[18] Morrow AL (1999). Neurosteroids mediate pharmacological effects of ethanol: a new mechanism of ethanol action?. Alcohol Clin. Exp. Res..

[19] VanDoren MJ (2000). Neuroactive steroid 3alpha-hydroxy-5alpha-pregnan-20-one modulates electrophysiological and behavioral actions of ethanol. J. Neurosci..

[20] Bernstein JE, Swift RM, Soltani K, Lorincz AL (1982). Antipruritic effect of an opiate antagonist, naloxone hydrochloride. J. Invest. Dermatol..

[21] Fujii M, Nabe T, Tomozawa J, Kohno S (2006). Involvement of skin barrier dysfunction in itch-related scratching in special diet-fed hairless mice. Eur. J. Pharmacol..

[22] Yamaguchi T (2001). Characterization of itch-associated responses of NC mice with mite-induced chronic dermatitis. J. Dermatol. Sci..

[23] Wetzel CH (1998). Functional antagonism of gonadal steroids at the 5-hydroxytryptamine type 3 receptor. Mol. Endocrinol..

[24] Reddy DS, Estes WA (2016). Clinical Potential of Neurosteroids for CNS Disorders. Trends Pharmacol. Sci..

[25] Carstens, E. & Kuraishi, Y. Animal models of itch: scratching away at the problem. Yosipovitch G., Greaves M. W., Fleischer A. B., McGlone F., Monticello (Eds), Itch: Basic Mechanisms and Therapy, *Marcel Dekker*, *NY*. 35–50 (2004).

[26] LaMotte RH, Shimada SG, Sikand P (2011). Mouse models of acute, chemical itch and pain in humans. Exp. Dermatol..

[27] Kasutani K (2014). Anti-IL-31 receptor antibody is shown to be a potential therapeutic option for treating itch and dermatitis in mice. Br. J. Pharmacol..

[28] Pauli-Magnus C (2000). Naltrexone does not relieve uremic pruritus: results of a randomized, double-blind, placebo-controlled crossover study. J. Am. Soc. Nephrol..

[29] Siemens W (2016). Pharmacological interventions for pruritus in adult palliative care patients. Cochrane Database Syst. Rev..

[30] Damianisch K, Rupprecht R, Lancel M (2001). The influence of subchronic administration of the neurosteroid allopregnanolone on sleep in the rat. Neuropsychopharmacology..

[31] Lancel M (1997). Allopregnanolone affects sleep in a benzodiazepine-like fashion. J. Pharmacol. Exp. Ther..

[32] Ebata T, Aizawa H, Kamide R, Niimura M (1999). The characteristics of nocturnal scratching in adults with atopic dermatitis. Br. J. Dermatol..

[33] Fujita H, Nagashima M, Takeshita Y, Aihara M (2014). Correlation between nocturnal scratch behavior assessed by actigraphy and subjective/objective parameters in patients with atopic dermatitis. Eur. J. Dermatol..

[34] Yosipovitch G (2002). Itch characteristics in Chinese patients with atopic dermatitis using a new questionnaire for the assessment of pruritus. Int. J. Dermatol..

[35] Zhu D, Wang MD, Bäckström T, Wahlström G (2001). Evaluation and comparison of the pharmacokinetic and pharmacodynamic properties of allopregnanolone and pregnanolone at induction of anaesthesia in the male rat. Br. J. Anaesth..

[36] Davies M (2003). The role of GABAA receptors in mediating the effects of alcohol in the central nervous system. J. Psychiatry Neurosci..

[37] Löscher W, Rogawski MA (2012). How theories evolved concerning the mechanism of action of barbiturates. Epilepsia..

[38] Olsen RW, Sieghart W (2009). GABA A receptors: subtypes provide diversity of function and pharmacology. Neuropharmacology..

[39] Winsky-Sommerer R (2009). Role of GABAA receptors in the physiology and pharmacology of sleep. Eur. J. Neurosci..

[40] Santhakumar V, Wallner M, Otis TS (2007). Ethanol acts directly on extrasynaptic subtypes of GABAA receptors to increase tonic inhibition. Alcohol..

[41] Saxena NC, Macdonald RL (1994). Assembly of GABAA receptor subunits: role of the delta subunit. J. Neurosci..

[42] Chandra D (2010). Prototypic GABA(A) receptor agonist muscimol acts preferentially through forebrain high-affinity binding sites. Neuropsychopharmacology..

[43] Korpi ER (2002). Altered receptor subtypes in the forebrain of GABA(A) receptor delta subunit-deficient mice: recruitment of gamma 2 subunits. Neuroscience..

[44] Akiyama T, Iodi Carstens M, Carstens E (2011). Transmitters and pathways mediating inhibition of spinal itch-signaling neurons by scratching and other counterstimuli. PLoS One..

[45] Cevikbas F (2017). Synergistic antipruritic effects of gamma aminobutyric acid A and B agonists in a mouse model of atopic dermatitis. J. Allergy Clin. Immunol..

[46] Chen L, Wang W, Tan T, Han H, Dong Z (2016). GABA(A) Receptors in the Central Nucleus of the Amygdala Are Involved in Pain- and Itch-Related Responses. J. Pain..

[47] Bonnet MP, Marret E, Josserand J, Mercier FJ (2008). Effect of prophylactic 5-HT3 receptor antagonists on pruritus induced by neuraxial opioids: a quantitative systematic review. Br. J. Anaesth..

[48] Kyriakides K, Hussain SK, Hobbs GJ (1999). Management of opioid-induced pruritus: a role for 5-HT3 antagonists?. Br. J. Anaesth..

[49] Ostadhadi S, Kordjazy N, Haj-Mirzaian A, Mansouri P, Dehpour AR (2015). 5-HT3 receptors antagonists reduce serotonin-induced scratching in mice. Fundam. Clin. Pharmacol..

[50] Tian B (2016). Peripheral and spinal 5-HT receptors participate in cholestatic itch and antinociception induced by bile duct ligation in rats. Sci. Rep..

[51] Agís-Balboa RC (2006). Characterization of brain neurons that express enzymes mediating neurosteroid biosynthesis. Proc. Natl. Acad. Sci. USA.

[52] Sanders KM, Akiyama T (2018). The vicious cycle of itch and anxiety. Neurosci. Biobehav. Rev..

[53] Ueda H, Amano H, Shiomi H, Takagi H (1979). Comparison of the analgesic effects of various opioid peptides by a newly devised intracisternal injection technique in conscious mice. Eur. J. Pharmacol..

[54] Hylden JL, Wilcox GL (1980). Intrathecal morphine in mice: a new technique. Eur. J. Pharmacol..

[55] Kaufman KR, Tanchuck MA, Strong MN, Finn DA (2010). Replacement with GABAergic steroid precursors restores the acute ethanol withdrawal profile in adrenalectomy/gonadectomy mice. Neuroscience..

[56] Belelli D, Bolger MB, Gee KW (1989). Anticonvulsant profile of the progesterone metabolite 5 alpha-pregnan-3 alpha-ol-20-one. Eur. J. Pharmacol..

[57] Bitran D, Hilvers RJ, Kellogg CK (1991). Anxiolytic effects of 3 alpha-hydroxy-5 alpha[beta]-pregnan-20-one: endogenous metabolites of progesterone that are active at the GABAA receptor. Brain Res..

[58] Singh S (2010). Allopregnanolone, the active metabolite of progesterone protects against neuronal damage in picrotoxin-induced seizure model in mice. Pharmacol. Biochem. Behav..

