# Supplementary material for: Brain allopregnanolone induces marked scratching behaviour in diet-induced atopic dermatitis mouse model
Source: Sci Rep. 2019 Feb 20;9:2364. doi: 10.1038/s41598-019-38858-3 (PMC6382911; doi:10.1038/s41598-019-38858-3)
Supplement: Supplementary file 3 — Supplemental video legends [file 41598_2019_38858_MOESM3_ESM.pdf]

Title

Brain allopregnanolone induces marked scratching behaviour in diet-induced atopic dermatitis mouse model

Author list and affiliation

Masanori Fujii[a,*], Sayaka Ohgami[a], Erika Asano[a], Takanori Nakayama[a], Takahiro Toda[a], Takeshi Nabe[a,b], Susumu Ohya[a,c]

[a]Department of Pharmacology, Division of Pathological Sciences, Kyoto Pharmaceutical University, 5 Nakauchi-cho, Misasagi, Yamashina, Kyoto 607-8414, Japan

[b]Laboratory of Immunopharmacology, Faculty of Pharmaceutical Sciences, Setsunan University, 45-1 Nagaotoge-cho, Hirakata, Osaka 573-0101, Japan

[c]Department of Pharmacology, Graduate School of Medical Sciences, Nagoya City University, 1 Kawasumi, Mizuho-cho, Mizuho, Nagoya 467-8601, Japan

*Corresponding author:

Masanori Fujii, Ph.D.

Department of Pharmacology, Division of Pathological Sciences, Kyoto Pharmaceutical University, 5 Nakauchi-cho, Misasagi, Yamashina, Kyoto 607-8414, Japan

Phone: +81-75-595-4668

E-mail: fujii@mb.kyoto-phu.ac.jp

Supplementary Information

Supplemental video-1 ALLO-induced scratching in atopic dermatitis-induced mice. ALLO (10 mg/kg, i.p.) was administered. This movie represents incessant scratching around 10 min after ALLO administration.

Supplemental video-2 ALLO-induced scratching in atopic dermatitis-induced mice. ALLO (5 μg/site, i.ci.) was administered. This movie represents incessant scratching around 10 min after ALLO administration.
